# Bridging the Rural–Urban Divide: Independent Pharmacies and Women’s Contraceptive Access

**DOI:** 10.3390/pharmacy14030081

**Published:** 2026-05-30

**Authors:** Amie M. Ashcraft, Anthony Peluso, Taylor Thompson, Amy Brenwalt, Sidney Sisson, Melody Phillips, Courtney S. Pilkerton, Charles D. Ponte

**Affiliations:** 1 Department of Family & Community Medicine, School of Medicine, West Virginia University, Morgantown, WV 26506, USA; taylor.thompson@hsc.wvu.edu (T.T.); amoyers1@hsc.wvu.edu (A.B.); ssisson@hsc.wvu.edu (S.S.); myphillips@hsc.wvu.edu (M.P.); cpilkerton@hsc.wvu.edu (C.S.P.); cdponte@hsc.wvu.edu (C.D.P.); 2 Department of Social & Behavioral Sciences, School of Public Health, West Virginia University, Morgantown, WV 26506, USA; apeluso@hsc.wvu.edu; 3 Department of Clinical Pharmacy, School of Pharmacy, West Virginia University, Morgantown, WV 26505, USA

**Keywords:** pharmacist, independent pharmacy, women’s health, reproductive health, contraceptive access

## Abstract

Independent community pharmacies serve as critical healthcare access points in rural areas, yet they consistently underperform chain pharmacies on contraceptive access measures. This narrative commentary draws on mystery caller studies, implementation research, and policy analyses to examine pharmacy-based contraceptive access in the United States (US). Using emergency contraception (EC) as a case study, we show that independent pharmacies stock EC at dramatically lower rates than chains (e.g., 14.6% vs. 76.3% in West Virginia), provide less accurate information about purchase requirements and timing, and impose more barriers to access. Because independent pharmacies account for 76.5% of pharmacies in rural areas, this disparity concentrates contraceptive inaccessibility in communities already facing the highest rates of unintended pregnancy, maternal mortality, and maternity care deserts. This pattern extends beyond EC to pharmacist-prescribed contraception and over-the-counter daily oral contraceptives. These disparities reflect systemic barriers, such as inadequate reimbursement, limited training infrastructure, and absence of corporate support, rather than failures of individual pharmacies. Drawing on implementation research and the success of West Virginia’s COVID-19 vaccination model, this paper proposes coordinated, sector-specific strategies to transform independent pharmacies from barriers into bridges for rural women’s contraceptive access.

## 1. Introduction

### 1.1. The Rural Healthcare Crisis

The United States (US) faces a severe maternal health crisis, particularly in rural communities. Approximately 36% of US counties are maternity care deserts where pregnant women have no or limited access to obstetric services (OB) [1]. Between 2011 and 2023, 293 rural hospitals stopped providing obstetric services, representing 24% of rural OB units nationwide [2]. Rural maternal mortality rates are 1.6 times higher than urban rates [3], and counties classified as maternity care deserts experience 36% higher maternal mortality rates even after adjusting for demographics and socioeconomic factors [4]. States with a high prevalence of maternity care deserts show 34% greater maternal mortality risk and 18% greater infant mortality compared to states with fewer deserts [5].

### 1.2. Contraceptive Access as an Intervention

Improving contraceptive access represents a critical intervention for addressing maternal health disparities given that an estimated 43.3% of US pregnancies are unintended [6]. Contraception lowers the risk of maternal deaths both by reducing the number of pregnancies and by preventing high-risk births, such as closely spaced and high parity pregnancies [7]. When women can plan their pregnancies, they are more likely to be in better health at the start of pregnancy, and more likely to seek prenatal care—both factors that directly influence maternal mortality [8,9]. For rural communities already facing the highest maternal mortality rates and maternity care deserts, ensuring access to contraception is not just a matter of reproductive choice but a potentially life-saving intervention.

### 1.3. The Pharmacy Opportunity

Community pharmacies that remain embedded in rural communities can serve as accessible alternatives to clinics that have closed. With approximately 70,000 pharmacies nationwide [10] compared to 17,000 publicly funded clinics, community pharmacies are uniquely positioned to fill gaps in contraceptive access [11]. Further, patients visit pharmacies nearly twice as often as primary care physicians [10,12,13], and pharmacists are recognized as among the most accessible healthcare providers, with 88.9% of the US population living within 5 miles of a pharmacy [10]. Independent pharmacies account for 76.5% of pharmacy locations in rural areas and offer deep community integration, trusted relationships with residents, and flexible hours that make them particularly accessible when traditional healthcare has disappeared [10,14].

Independent pharmacies have demonstrated the potential for expanded services when given appropriate support. During the COVID-19 pandemic, West Virginia became a national leader in vaccination rates by opting out of the federal partnership with CVS and Walgreens, instead relying on its network of independent pharmacies to leverage their existing community relationships and infrastructure to rapidly deliver vaccines to rural and underserved populations [14]. The success of this model demonstrated that independent pharmacies could mobilize effectively for public health interventions when barriers are removed and appropriate support is provided.

### 1.4. The Paradox

These strengths make independent pharmacies essential with approximately 15.1 million Americans depending on them exclusively for pharmacy services [15]. Yet despite this critical role, independent pharmacies consistently underperform chain pharmacies on contraceptive availability and access [14,16,17,18,19,20,21,22,23,24,25,26].

This gap affects the most vulnerable populations. Rural residents are six times more likely than urban residents to rely solely on independent pharmacies (16.3% vs. 2.6%), and low-income households depend on them more than high-income households (5.8% vs. 4.0%) [15]. These same populations already face elevated risks of pregnancy complications, preterm birth, and death [27]. The gap is concentrated geographically with the residents of West Virginia, Mississippi, Arkansas, Louisiana, Tennessee, and Montana relying heavily on independent pharmacies as primary access points. Simultaneously, these patients experience the nation’s poorest reproductive health outcomes—demonstrating that where contraceptive access is most needed, it is least available [10,15,27,28]. More than 1.5 million Appalachian women live in a contraceptive desert [29], and these contraceptive deserts overlap geographically with maternity care and pharmacy deserts, compounding the inaccessibility in the same communities [1,15].

### 1.5. Expanding Contraceptive Access at Pharmacies

Addressing independent pharmacies’ contraceptive access gap has become more urgent as pharmacies take on a greater role in contraceptive services. In July 2023, the Food and Drug Administration (FDA) approved Opill, the first over the counter (OTC) daily oral contraceptive available without prescription or age restrictions. At the same time, states are increasingly approving pharmacist prescription of contraception, with 30 states plus the District of Columbia implementing these laws [30]. These policy changes position pharmacies in underserved areas as game changers for contraceptive access.

Early data suggest that OTC contraception availability to uninsured and rural populations is increasing, but most women do not know it exists [31,32]. Among contraceptive users, 32.5% chose OTC options. Compared with prescription users, OTC users were far more likely to be uninsured (31.6% vs. 3.5%) and to reside in rural areas (14.4% vs. 8.4%) [32]. However, only 26% of reproductive-age women are aware of Opill, with even lower awareness among those who most need access, including uninsured, rural, and women of color [33]. Similarly, in a rural California community, only 31% of reproductive-age women were aware that pharmacists could prescribe contraception, yet 57% expressed interest once informed [34]. Whether these policy changes narrow or widen the access gap depends on whether independent pharmacies participate.

### 1.6. The Purpose of This Paper

This paper uses emergency contraception (EC) as a case study for examining contraceptive access disparities between independent and chain pharmacies. EC is useful for this purpose for three reasons. First, since 2013 levonorgestrel EC has required no age restrictions, no prescription requirements, and only straightforward dosing and counseling [35,36]. These minimal regulatory requirements suggest that access barriers arise from pharmacy operations and implementation challenges and not the law itself. Second, EC is time-sensitive and less effective when delayed, making access problems quickly apparent. Third, mystery caller research has documented chain pharmacies outperforming independent pharmacies on EC availability and access as well as accuracy of information provided and willingness to order EC [16,18,20,24,26,37,38,39].

The American Pharmacists Association and Contraceptive Access Initiative Summit in 2024 brought together 26 organizations to identify barriers affecting all pharmacy-based contraceptive services [40]. Their findings indicated that the challenges of reimbursement complexity, training costs, workflow integration, and liability concerns affect all pharmacies, but create disproportionately larger challenges for independent pharmacies with thinner profit margins, fewer staff, less corporate support, and greater isolation. These findings are consistent with research showing that independent pharmacists cite lack of insurance reimbursement, liability concerns and absence of corporate mandates as primary barriers to implementing expanded contraceptive services [21,41]. These barriers show that EC access problems reflect broader operational challenges as opposed to issues with a single product.

Despite extensive documentation of EC access barriers, major research gaps remain for newer contraceptive services. While some data on pharmacist-prescribed contraception shows that chain pharmacies are more likely to offer these services than independent pharmacies [22,42], we lack comprehensive studies comparing service quality, counseling accuracy, and patient outcomes across pharmacy types. Similarly, while early Opill stocking data shows the familiar pattern of chain pharmacies providing better access than independent pharmacies [25], we lack studies examining pricing, staff knowledge or counseling quality. Until more systematic research exists across all newer contraceptive services, EC remains our clearest evidence base for understanding how independent and chain pharmacies differ in their approach to contraceptive access.

This paper is a narrative commentary rather than a systematic review. We drew on several types of evidence, including US-based mystery caller studies of pharmacy contraceptive access, implementation research on pharmacist-prescribed and OTC contraception, policy analyses, and qualitative studies of pharmacist perspectives. We identified studies through targeted searches of PubMed, Google Scholar, and relevant policy and professional organization sources, with a focus on research published in the last decade and on work that compared independent and chain community pharmacies. We selected EC as a case study for the reasons described above: its regulatory simplicity, time-sensitivity, and the strength of the mystery caller evidence base. Together, these characteristics make it possible to attribute access barriers to pharmacy operations as opposed to the law or to product complexity. From this evidence, we identify patterns across studies and propose sector-specific actions to close the gap between independent and chain pharmacies.

The goal of this paper is to propose evidence-based solutions that help turn independent pharmacies from barriers to bridges in rural women’s contraceptive access. The effort will require action from independent pharmacies, professional organizations, policymakers, healthcare systems, and researchers. Now, more than ever, we need to translate evidence into action.

## 2. The Evidence—Emergency Contraception as a Case Study

EC provides a window into how independent pharmacies handle OTC contraceptive access. Despite federal policy making levonorgestrel EC (“Plan B”) available without restrictions since 2013, research reveals persistent barriers at independent pharmacies with availability, accessibility, and information accuracy. These barriers are largely absent from chain pharmacies, and the pattern is consistent across diverse US geographic regions and time periods.

### 2.1. The National Pattern: Availability, Accessibility, and Information

The most fundamental barrier is product availability. In West Virginia, a recent study revealed that only 14.6% of independent pharmacies stocked EC compared to 76.3% of chain pharmacies [16]. This pattern repeats nationwide: in a Colorado study, 57.5% of independent pharmacies versus 90.4% of chains stocked EC [18]; in a southwestern Pennsylvania study, only 48% of independent pharmacies had EC available compared to 83% of chains, and the gap was wider for on-the-shelf access, with 16% of independents versus 61% of chains [23]; and a four-state study of Arizona, California, New Mexico, and Utah documented the same pattern [24].

Even when stocked, EC is often functionally inaccessible at independent pharmacies. Complete accessibility—that is, availability on store shelves without stated age, identification, or prescription requirements—existed in only 2.7% of West Virginia independent pharmacies compared to 47% of chains [16]. Colorado found complete accessibility at 10% of independent pharmacies versus 25% of chains, with 41.6% keeping medication behind the counter [18]. In Pennsylvania, 70% of pharmacies that stocked EC on the shelf kept it in locked boxes requiring staff assistance [23].

Staff knowledge often compounds these barriers. In West Virginia, only 32.9% of independent pharmacy staff provided accurate information about EC timing for effectiveness compared to 62.9% of chain pharmacy staff [17], and misinformation about point-of-sale requirements was pervasive. Independent pharmacy staff were significantly more likely to incorrectly tell callers that a prescription, identification, or parental consent was required, despite OTC status having been in place for years [17]. A systematic review confirmed that inaccurate information about regulations, mechanism of action, and administration persists across pharmacy settings, with independent pharmacies showing particularly low knowledge [43].

### 2.2. Rural Impact and Geographic Consistency

The independent pharmacy access gap creates compounded disadvantages in rural areas where independent pharmacies comprise a larger share of pharmacy services. West Virginia GIS mapping shows chain pharmacies cluster along highways and in populated areas, while rural communities rely predominantly on independent pharmacies with dramatically lower contraceptive availability [17]. National GIS analyses confirm this geographic distribution: in large urban areas, chains represent 62.8% of pharmacies, but in rural areas, 76.5% are independent pharmacies [10]. When controlling for pharmacy type, studies across Colorado, Arizona, California, New Mexico, and Utah found that EC availability was significantly associated with pharmacy type but not with rural versus urban location [18,24]. Rural areas face access challenges to EC not because they are rural, but because they are more likely to be served by independent pharmacies.

### 2.3. Emergency Contraception as Predictor: Evidence from Other Contraceptive Services

If the access gap for a straightforward OTC product like EC is this wide, the implications for more complex pharmacy-based contraceptive services requiring patient screening, counseling, and billing are concerning. The independent-chain disparity extends beyond EC to other pharmacy-based contraceptive services, indicating that these are not problems with the specific products but rather reflect how independent and chain pharmacies operate differently. When Opill launched in March 2024, only 25% of independent pharmacies in Texas stocked it compared to 82% of standalone chain pharmacies [25]. State-level data on pharmacist prescribing shows similar patterns. In Utah, chains represented 80% of pharmacies providing contraceptive prescribing services one year after implementation [22]. Qualitative research reveals that chain pharmacies benefit from corporate policies requiring all pharmacists to complete training and implement services, while independent pharmacies must rely on individual champions to drive implementation [21,44]. The consistency of this pattern for EC, pharmacist-prescribed services, and OTC oral contraceptives demonstrates systematic differences in how independent and chain pharmacies approach pharmacy-based contraceptive access.

## 3. Barriers Facing Independent Pharmacies

Independent pharmacies consistently provide less access to contraception than chain pharmacies, but this pattern reflects systemic barriers rather than individual pharmacies failing [21,44]. The barriers to providing these services reinforce one another: financial constraints limit the capacity for training and staffing, which leads to knowledge gaps, which shape what products pharmacies stock and the services they offer, all in settings where one pharmacist’s decision can determine whether an entire community has access. Understanding this cascade is essential for developing solutions that address the causes rather than the symptoms.

The cascade begins with financial constraints. Independent pharmacies operate on thin margins, with reimbursement rates that often fail to cover the cost of dispensing, let alone clinical services [21]. In most states, pharmacists cannot bill insurers for contraceptive consultation time, forcing them to choose between offering free services or implementing out-of-pocket fees that patients in underserved areas may not be able to afford [21,45]. Many independent pharmacists see the value in offering more clinical services to stay competitive, but the upfront costs are hard to justify when the financial return is uncertain. Chain pharmacies absorb these costs across thousands of locations, while independent pharmacies cannot.

Financial constraints directly limit training and knowledge. Without corporate-mandated continuing education, independent pharmacy staff must identify and pay for their own training on contraceptive provision and counseling, a cost in both money and time that not all can afford [21,41]. PharmD curricula dedicate minimal time to contraception, averaging less than one hour on EC across US programs [46]—and may not prepare all graduates to confidently counsel on EC timing, pharmacist-prescribed contraception protocols, or OTC options like Opill [47,48]. In chain pharmacies, corporate mandates require training completion before service implementation, while in independent pharmacies, training depends on individual initiative [45].

Knowledge gaps and financial constraints together shape inventory decisions. Independent pharmacies stock medications based on local demand, and for products like EC that may have low turnover in one or another community, the investment of keeping stock on hand may not seem justifiable with already narrow margins. Notably, no known qualitative research has systematically examined independent pharmacists’ reasons for not stocking EC or other contraceptive products. Given how consistently the stocking gap appears across studies, this is an area that future research should address. When Opill launched, independent pharmacies did not have the bulk purchasing deals and manufacturer connections that enabled chain pharmacies to stock the product right away [25].

Finally, independent pharmacies face community pressures that chain policies do not. When there is no corporate policy requiring a product to be on the shelf, the decision falls to the individual pharmacist-owner, including whether personal or moral beliefs justify not stocking contraceptive products at all. In a West Virginia study, four independent pharmacies (2%) explicitly cited personal or moral reasons for not stocking EC compared to no chain pharmacies [16]. Even when moral objections are not given, the absence of corporate policy means that stocking decisions rest with individual pharmacist-owners who are embedded in their communities in ways that chain pharmacists are not, and research on so-called “pharmacist gatekeeping” shows that this organizational context shapes how personal and community values influence what products are stocked and sold [49].

Of course, independent pharmacies are not all the same. They vary considerably in staffing, technician support, access to continuing education, workflow integration, and the scope of clinical services they choose to offer. Some independent pharmacies have robust clinical services and have active partnerships with local providers, while others operate with minimal staff and have limited capacity to take on services beyond their existing workload. This variability means that the barriers described above affect different pharmacies differently, and one-size-fits-all solutions designed around chain pharmacy infrastructure are unlikely to work across varied independent pharmacy settings. Tailored support rather than uniform mandates will be critical. Independent pharmacies with more existing infrastructure may need minimal assistance to expand contraceptive services, while those with less infrastructure will need more support around staffing, training, and reimbursement before services can be expanded.

Across all these barriers, the consistent finding is that independent pharmacists overwhelmingly support expanded contraceptive services but report lacking the resources to implement them [21,41,50,51,52]. Independent pharmacists are not failing their communities. Rather, the system in which they operate was not built to support them.

## 4. Evidence-Based Solutions to Turn Barriers into Bridges

The barriers are substantial, but implementation research demonstrates that targeted interventions can transform them into bridges for contraceptive access. A multistate qualitative study using the Consolidated Framework for Implementation Research (CFIR) identified key steps, facilitators, and barriers across ten states implementing pharmacist-prescribed contraception, documenting what worked and what did not across diverse state contexts [44].

Several CFIR domains are particularly relevant to independent pharmacies. The outer setting, such as the policy and reimbursement environment that surrounds a pharmacy, determines whether expanded contraceptive services are even financially or legally possible. The inner setting, including a pharmacy’s staffing, workflow, resources, and day-to-day climate, determines whether a pharmacy can offer those services in practice. Finally, individual-level factors, such as pharmacist knowledge, training, and personal beliefs, shape whether services are offered consistently when no corporate policies require it. The barriers described in the previous section also fall into these categories: reimbursement and regulation come from outside the pharmacy, training and staffing gaps come from inside it, and variability in pharmacist knowledge and willingness come from the individual level. Each kind of barrier requires a different kind of response. As such, the solutions below are organized by sector, beginning with interventions most likely to drive change across the entire system.

### 4.1. For Policymakers: Reimbursement and Regulatory Clarity

The most critical barrier to address is reimbursement. Across multiple studies, community pharmacists consistently identify lack of payment for services, time constraints, and liability concerns as the primary obstacles to implementing contraceptive services, with reimbursement being the most cited barrier regardless of state context or pharmacy type [21,41,44,45,50,51,52,53]. Without a financially sustainable service model, independent pharmacies cannot absorb the costs of training, workflow changes, and consultation time. State Medicaid programs can help support this effort. North Carolina’s Medicaid program enables beneficiaries to obtain Opill at retail pharmacies without a prescription or cost-sharing, and pharmacies can bill Medicaid directly.

As of early 2026, at least ten states have enacted legislation or taken executive action related to OTC contraceptive coverage in state-regulated plans or state Medicaid programs [54,55]. Research has found that states with reimbursement mechanisms in place were consistently further along in implementation than those without [44]. The expanding evidence base supports expanded pharmacy-based contraceptive services beyond oral methods. A recent systematic review found that patients, pharmacists, and other health care professionals view pharmacist-administered injectable contraception favorably, identifying it as a feasible and accessible option that could further address contraceptive access gaps, particularly in rural communities [56]. Yet, reimbursement alone is not sufficient if pharmacies cannot navigate the rules for providing and billing these services.

Regulatory clarity matters as much as reimbursement. Inconsistent federal guidance on OTC contraceptive coverage has created confusion among insurance plans, pharmacies, and patients about what is covered and how to bill for it [57]. Unclear or inflexible state regulations can stall implementation even after prescribing authority has been granted [58,59]. The state of Washington addressed this by developing a pharmacist toolkit with step-by-step billing templates, FAQs for both patients and pharmacy staff, and clear guidance that OTC contraception is covered at no cost [31,60]. This is information that chain pharmacies receive through internal corporate communication but that independent pharmacies lack.

West Virginia’s COVID-19 vaccination program demonstrates what is possible when states invest in independent pharmacy partnerships. When the state provided independent pharmacies with direct access to vaccines, clear guidance on answering patient questions and concerns, and streamlined administrative processes, these pharmacies led the nation in vaccination rates [14,61]. The effort succeeded, in part, because the state removed bureaucratic barriers, provided logistical support, and treated independent pharmacies as partners in the distribution plan. Independent pharmacies were uniquely positioned for this role because their community trust, local knowledge, and existing patient records gave them an infrastructure that clinics and chain pharmacies could not replicate. This proved especially valuable in rural areas where populations are geographically dispersed.

### 4.2. For Professional Organizations: Training Infrastructure and Cross-State Learning

Supportive policies are ineffective if pharmacists are not prepared to provide the services needed. Pharmacist willingness already exists, with 65% of US community pharmacists interested in prescribing hormonal contraception, motivated primarily by the opportunity to expand their clinical role with patients [41]. What is lacking is the infrastructure to translate interest into action, particularly for independent pharmacies without corporate training infrastructure. Multiple low-cost and free training options have emerged. Oregon State University offers American Council for Pharmacy Education (ACPE)-accredited comprehensive contraceptive education programs accepted by boards of pharmacy in multiple states [62]. The World Health Organization launched a free online course specifically designed to build capacity among pharmacy workers through case studies and practice exercises [63]. The American Pharmacists Association offers advanced training in hormonal contraception with the goal of expanding access to vulnerable communities [64]. The Birth Control Pharmacist website (birthcontrolpharmacist.com) provides a centralized hub with ACPE-accredited training, clinical resources, patient education materials, and state-specific implementation toolkits.

The availability of training alone is not sufficient. Implementation research shows that state pharmacy associations and state-level champions were critical facilitators of successful implementation [44,58]. States that networked with other states to adapt existing protocols rather than developing them from scratch were more likely to succeed [44,58]. Organizations like the National Alliance of State Pharmacist Associations enable this cross-state learning. The challenge is that most states lack centralized programs to advertise training opportunities, leaving independent pharmacists to find resources on their own [44,59]. Professional organizations are uniquely positioned to close this gap.

Schools of Pharmacy represent an underutilized partner in this infrastructure. Pharmacy students are often members of the reproductive-age population most affected by contraceptive access gaps. They bring both content knowledge and credibility as peer educators when engaging practicing pharmacists, preceptors, and pharmacy staff, especially during advanced pharmacy practice rotations. Strengthening contraceptive content in PharmD curricula—currently averaging less than one hour on EC across US programs [46]—would build a workforce prepared to implement these services from their first day of practice. Student rotations placed in independent pharmacies can simultaneously raise awareness of pharmacist-prescribed contraception and OTC options like Opill, support preceptor education, and expose students to rural and underserved practice settings where workforce shortages are most concentrated. Student pharmacist organizations can extend this reach through community outreach that addresses the awareness gap documented among reproductive-age women, especially those from uninsured, rural, and minority populations [33,34]. Engaging Schools of Pharmacy as active partners can help bring contraceptive services to the independent pharmacies that most need them.

### 4.3. For Healthcare Systems: Partnerships and Referral Networks

Healthcare systems can support contraceptive access in independent pharmacies regardless of whether a state has authorized pharmacist prescribing. In states where pharmacists cannot prescribe independently, collaborative practice agreements offer a tested alternative. Purdue University’s campus pharmacy implemented contraceptive prescribing via a collaborative drug therapy management agreement with the student health center, enabling pharmacists to prescribe pills, patches, rings, injections, and emergency contraception [65]. This shows that pharmacies can offer prescribing services through formal partnerships with health systems, even in states that have not passed prescribing legislation.

In all states, healthcare systems can strengthen independent pharmacy services in many ways. Formalized referral networks allow pharmacists to connect patients to providers for long-acting reversible contraception, which requires provider insertion but benefits from pharmacist counseling about methods and referral [66,67]. Most states allowing pharmacists prescriptive authority require them to notify patients’ primary care providers after a contraceptive encounter [58], but this notification is most useful when health systems can effectively receive it and coordinate follow-up care [59]. Sharing electronic health records (EHR) or patient information with pharmacy partners can reduce fragmentation and ensure continuity of care across settings, though EHR sharing between pharmacies and health systems remains in the early stages of development [68].

Several practical barriers make these partnerships challenging to implement. EHR interoperability between independent pharmacies and systems is often limited, and many independent pharmacies use dispensing software that does not communicate easily with hospital or clinic systems [68]. Documentation standards for pharmacist-provided contraception encounters vary by state and are not always compatible with how health systems record care. This can create gaps in the patient record and make billing more complicated. Further, collaborative practice agreements require legal review, malpractice considerations, and clear delineation of pharmacist scope [69]. All this can be resource-intensive for small independent pharmacies without legal or compliance staff. Finally, communications between pharmacies and health systems often utilize fax or paper records, particularly in rural areas where broadband access is limited [70]. Without resolution of these challenges, even strong partnerships can stall.

Despite these challenges, one of the most straightforward ways to turn independent pharmacies from barriers into bridges is for health systems to incorporate them into their contraceptive care infrastructure, extending the health system’s reach into communities where clinic access is limited. Independent pharmacists encountering patients with complex medical histories also benefit from access to clinical backup and consultation with physicians and specialists—the kind of support that health system partnerships could provide.

### 4.4. For Independent Pharmacies: Practical Implementation

Independent pharmacies have a critical role to play in putting the aforementioned interventions into practice, and the available evidence is encouraging. In California, 97% of patients who received pharmacist-prescribed contraception reported overall satisfaction, and the most common reasons patients sought pharmacy-based contraception were speed and convenience [71], two things that independent pharmacies in rural communities are well-positioned to provide.

Providing these services effectively requires operational preparation. Implementation studies show that contraceptive consultations average 20 min, but efficiency improves with experience and standardized protocols [65]. States that allowed pharmacy school graduates to prescribe without additional training requirements made it easier for new pharmacists to begin offering these services [44]. Expanding pharmacy technician roles to handle administrative tasks during contraceptive consultations can also alleviate pharmacist workload [44]. Pharmacies also need adequate private consultation space for sensitive reproductive health conversations, a practical requirement that multiple studies identify as a barrier to implementation [51,67]. Early adopters of pharmacist-prescribed contraceptive services found that success required more efficient workflows. It also depended on community outreach, sustainable funding plans, and strategies for managing staff turnover [45], challenges that are especially relevant for independent pharmacies without corporate marketing or human resources support.

There is support for independent pharmacies to navigate these challenges. Pharmacies joining networks like the Community Pharmacy Enhanced Services Network (CPESN) can share infrastructure, negotiate collectively with payers, and access peer support that reduces the isolation identified as a key barrier to expanding contraceptive services [21,44,72]. Maryland’s CPESN chapter received grant funding to support contraceptive service implementation through partnerships with Schools of Pharmacy and pharmacy resident training programs [72].

### 4.5. For Researchers: Priority Gaps

The evidence base for pharmacy-based contraception is growing, but little of it focuses on independent pharmacies specifically. Oregon’s pharmacist-prescribed contraception program averted an estimated 51 unintended pregnancies and saved $1.6 million within the first two years of implementation among Medicaid beneficiaries alone [73]. Additionally, 74% of women receiving pharmacist-prescribed contraception were new contraceptive users who had not used any prescribed method in the prior month, suggesting that the program was reaching women who were not already using prescribed contraception [74]. Yet most participating pharmacies were chains, and no comparable cost effectiveness data exists for independent pharmacy interventions. This gap extends beyond cost-effectiveness studies. We also need systematic comparisons of service quality, counseling accuracy, and patient outcomes between independent and chain pharmacies after implementing support interventions [16,18,22,23,25,42]. Notably, no published qualitative research has systematically examined independent pharmacists’ reasons for not stocking EC or other contraceptive products despite the consistency of the stocking disparity. Future research should focus on what kind of support actually helps independent pharmacies expand contraceptive services [44,45]. A key question is whether the barriers documented for EC disappear when pharmacies receive adequate training, reimbursement, and infrastructure support, or whether new ones emerge.

### 4.6. Considerations and Tradeoffs

Beyond these sector-specific actions, expanding pharmacy-based contraceptive access raises broader considerations worth acknowledging. Pharmacy-based contraceptive services are not a substitute for comprehensive reproductive and preventive care. Traditional primary care and reproductive health visits also offer cervical cancer screening, sexually transmitted infection (STI) testing and treatment, breast exams, and counseling on a broad array of reproductive issues. Women who obtain contraception primarily through a pharmacy may have fewer touchpoints with their providers over time. This is a particular concern in rural and underserved areas where pharmacy access may already be replacing traditional healthcare that has disappeared with clinic and hospital closures and provider shortages.

Fragmentation of care is a related concern. If pharmacist-provided contraceptive encounters are not communicated to primary care providers, the resulting gaps in the patient record can affect medication management, chronic disease care, and preventive services. Most states with pharmacist prescribing require notification of the patient’s provider [58] but as mentioned above, the systems that make this notification meaningful are not consistently in place.

These concerns are not arguments against expanding pharmacy-based contraceptive access, but they do argue for implementing them carefully. Pharmacist counseling could routinely include information about cervical cancer screening, STI testing and other preventive services. Pharmacies themselves can serve as repositories for related supplemental or additional written and/or digital healthcare information. Pharmacists could also provide referrals to primary care for women who do not have an established provider. Communication between pharmacies and health systems should be strengthened along with service expansion. Policy efforts should frame pharmacy access as a complement to (and not a replacement for) comprehensive reproductive healthcare. With careful design, expanded pharmacy-based contraceptive services can expand access without compromising continuity of care.

## 5. From Evidence to Action

Filling the research and infrastructure gaps described above will be essential for designing effective, evidence-based support for independent pharmacies, but the window for action is narrowing. Between 2003 and 2021, independently owned pharmacies in the most rural counties declined by 16.1%, and 630 communities lost their last retail pharmacy entirely [15,75]. Each closure removes not just a dispensing site, but a potential access point for contraception, counseling, and referral in communities that have already lost clinics, hospitals, and obstetric services. The convergence of maternity care deserts, contraceptive deserts, and pharmacy deserts in the same geographic areas means that the independent pharmacy is among the few remaining healthcare access points for millions of women. Losing it deepens every other gap.

The evidence presented in this paper is consistent across studies and geographic regions. Independent pharmacies provide less contraceptive access than chain pharmacies because they lack the financial resources, training infrastructure, and corporate support systems that chain pharmacies rely on. Each independent pharmacist must figure out reimbursement, training, regulatory compliance, and workflow changes on their own while keeping the pharmacy running.

But the evidence also points to concrete next steps. Sustainable reimbursement, accessible training, clear regulatory guidance, health system partnerships, and pharmacy networks that reduce isolation are strategies already being implemented in states across the country, and the early results are promising. West Virginia’s COVID-19 vaccination program demonstrated what independent pharmacies can accomplish when the state removes bureaucratic barriers, provides logistical support, and leverages the community trust that these pharmacies have been building for decades. The same model can work for contraception.

What is needed now is coordinated action. Policymakers must prioritize reimbursement and regulatory clarity so that independent pharmacies can afford to offer contraceptive services. Professional organizations must build the training and peer support infrastructure that corporate pharmacy chains provide internally. Healthcare systems must incorporate independent pharmacies into their contraceptive care networks rather than treating them as separate, disconnected entities. Research initiatives should incorporate the often overlooked independent pharmacies, beginning with why they do not stock contraceptive products and ways to improve product availability. And independent pharmacies themselves must engage with available resources, join collaborative networks, and participate as active partners in expanding contraceptive services.

Every independent pharmacy that stocks emergency contraception, trains staff on accurate counseling and participates in pharmacist-prescribed contraception programs moves one more rural community from desert to access point. Turning independent pharmacies from barriers into bridges for contraceptive access will require long-term investment from every sector. The infrastructure is there, pharmacists are willing, and patients want these services. What is missing is the coordinated effort to bring them together before the pharmacies that can deliver these services disappear.
